# Editorial: Recognition and critical social research

**DOI:** 10.3389/fsoc.2026.1924183

**Published:** 2026-07-30

**Authors:** Gottfried Schweiger, Luiz Gustavo Da Cunha De Souza

**Affiliations:** 1 University of Salzburg, Salzburg, Austria; 2 Universidade Federal de Santa Catarina, Florianópolis, Brazil

**Keywords:** Axel Honneth, critical social research, critical theory, recognition, social theory, sociology, social critique

The theory of recognition has, since the early 1990s, developed into one of the most productive paradigms in the field of social philosophy and critical social theory. The aim of this Research Topic in *Frontiers in Sociology* is to discuss this paradigm in a range of implications—conceptual, methodological, and empirically applied—through attempts of actualizing, criticizing, and make use of the concept of the concept of recognition and its vocabulary to contemporary social-theoretical issues. The central question is what recognition as a concept can contribute to critical social research, which limitations become visible in the process, and how these might be overcome. The Research Topic brings together contributions that partly address fundamental theoretical questions of recognition theory and partly test its application to concrete social fields.

The theoretical foundations on which the contributions are built are well-known. Since the work of Axel Honneth, recognition has no longer been merely a philosophical concept but a tool of social analysis used to describe and criticize social conflicts, inequalities, and pathologies. Honneth famously distinguishes three forms of recognition—love, rights, and social esteem—each corresponding to specific spheres of social life and whose denial leads to different forms of damage to personal identity (Honneth, 1996). Subsequently, he unfolds this paradigm in a full-scale theory of modern ethical life (Honneth, 2014), and more recently proves its consequences as am ambitious attempt to normatively reform the contemporary capitalist division of labor (Honneth, 2023). However, neither his basic model, nor its consequences remain uncontested, and it is precisely the critical engagement with them that holds this Research Topic together.

The articles gathered here could be assembled in three loosely defined groups. The first one, is primarily dedicated to methodological issues internal to the paradigm of recognition, specifically of Honneth's theory. Hirvonen's contribution addresses questions concerning the range of recognition and the role of mediating institutions; Rutherford's deals with the ideological forms of recognition and its connection to systemic forms of development; Visser's article advances from Honneth recognition theory into his normative reconstruction to suggest a wider dialog with the critical theory of organizations. What initially connects this three articles is the central importance they all attribute to the institutional frame of recognition but a closer look reveals that the three authors place special attention to the concept of power and how it can be accommodated within a critical theory of recognition.

The second cluster of articles presented in this Research Topic is not entirely disconnected from methodological issues; however, the contributions by Manterys, Schweiger and de Nardis, each of these authors in his own way, take the paradigm of recognition as a starting point for conceptual reflections on broader issues. All of those articles convincingly discuss questions of power within recognition and its institutions, to be sure; but they all primarily prove if recognition is a conceptually adequate mean to deal with connected social-theoretical issues. Manterys' turns to the concept of social pathology and undertakes an attempt to reconstruct its genealogy and make it fruitful for a transdisciplinary analysis of relations of recognition; Schweiger argues that recognition is structured not only socially but also temporally—when recognition is granted or withheld, how long this lasts, and how it is embedded in biographical regimes are normatively relevant questions—and shows the importance of this dimension to social welfare policies against poverty and precarization; de Nardis, finally, addresses the tension between the political consequences of a theory of recognition's implicit concept of freedom and the quest for emancipation.

A third group, and again not totally disconnected from the previous one's, could be divided in two internal blocks. In Pilapil's discussion of the Moro conflict in Mindanao, Philippines and in Ishimatsu's discussion of the practice of *tabunka-kyosei* (multicultural coexistence/colinving) in Japan are found examples of potentials and limits for an applied ethics of recognition. Whereas, Pilante delves into the very sensitive issue of the use of violent resistance against injustice and the just causes that could allow for it, Ishimatsu challenges the diffuse practices of inclusion and respect associated with multiculturalism through a reading of Honneth's theory of recognition as a critical standard. Both contributions, ultimately, connect the paradigm of recognition and its critical potential to empirical conditions of its application but do that from a rather theoretical viewpoint. On a second block, Parada-Ulloa et al. develop a theoretical rearticulation of the knowledge–power nexus in late modern societies. They show that classical theories of power are insufficient to explain how expert knowledge in societies shaped by expertocracy and algorithmic mediation simultaneously functions as legitimation, normalization, and a technology of governance. In addition, Mollès and Parada-Ulloa, together with Parada-Ulloa et al., examines critical pedagogies as fields of resistance in which questions of emancipation and democracy are negotiated.

The contributions assembled here make clear that recognition theory is a vibrant and at the same time contested field of research. They illuminate it from both systematic and empirical perspectives, from philosophy as well as sociology, and they combine normative reflection with analyses of concrete social conditions. Concepts like power and institutions, perspectives like that of multiculturalism or education, and subjects like social pathologies, freedom, and welfare policies are all covered in this Research Topic and the range of reflection they represent is also a testimony of how the paradigm of recognition still invites reflections about its internal architecture, its conceptual utility, and its practical value. Thus, this Research Topic represents—at least it is to be hoped—a contribution to critical social research that achieves both theoretical reflexivity and societal relevance.
